# Impact of timing of computed tomography staging and patient factors on the detection of ‘true’ cN+ bladder cancer

**DOI:** 10.1111/bju.16851

**Published:** 2025-07-09

**Authors:** Markus von Deimling, Marc Furrer, Alberto Bianchi, Renate Pichler, Moritz Maas, Karl H. Tully, Mattia Longoni, Laura S. Mertens, Jacob Taylor, Francesco del Giudice, Roger Li, Andrea Gallioli, Simone Albisinni, Felice Crocetto, Maud Velev, Luca Afferi, Andrea Mari, Ekaterina Laukhtina, Jakob Klemm, Nirmish Singla, Margit Fisch, Philippe E. Spiess, Yair Lotan, Marco Moschini, Peter C. Black, Alessandro Antonelli, Bernhard Kiss, Shahrokh F. Shariat, Benjamin Pradere, CLIPOLY study group collaborators, Luca Antonelli, Mara Bacchiani, Ronan Flippot, Abhinav Khanna, Elisabeth Maier, Gautier Marcq, Andrea Minvervini, Keiichiro Mori, Maximilian Pallauf, Nicolas Penel, Julien Sarkis, Francesco Soria, Guus A.H. Tendijck, Noor van Ginkel, Solomon Woldu

**Affiliations:** ^1^ Department of Urology University Medical Center Hamburg‐Eppendorf Hamburg Germany; ^2^ Department of Urology Eberhard Karls University Tübingen Tübingen Germany; ^3^ Department of Urology and Neurourology, Marien Hospital Herne Ruhr‐University Bochum Herne Germany; ^4^ Department of Urology, Comprehensive Cancer Center Medical University of Vienna Vienna Austria; ^5^ Karl Landsteiner Institute of Urology and Andrology Vienna Austria; ^6^ Department of Urology, Comprehensive Cancer Center Innsbruck Medical University of Innsbruck Innsbruck Austria; ^7^ Department of Urology, University Hospital of Bern University of Bern Bern Switzerland; ^8^ Department of Urology Solothurner Spitäler AG Olten and Solothurn Switzerland; ^9^ Department of Urology Luzerner Kantonsspital Luzern Switzerland; ^10^ Department of Urology University of Verona, Azienda Ospedaliera Universitaria Integrata Verona Italy; ^11^ Department of Urology Urological Research Institute, Vita‐Salute San Raffaele Milan Italy; ^12^ Department of Maternal Infant and Urologic Sciences “Sapienza” University of Rome, Policlinico Umberto I Hospital Rome Italy; ^13^ Urology Unit, Department of Surgical Sciences, Tor Vergata University Hospital University of Rome Tor Vergata Rome Italy; ^14^ Department of Neurosciences, Reproductive Sciences and Odontostomatology University of Naples “Federico II” Naples Italy; ^15^ Unit of Oncologic Minimally‐Invasive Urology and Andrology, Department of Experimental and Clinical Medicine, Careggi Hospital University of Florence Florence Italy; ^16^ Department of Urologic Sciences University of British Columbia Vancouver British Columbia Canada; ^17^ Department of Urology The Netherlands Cancer Institute Amsterdam The Netherlands; ^18^ Department of Urology University of Texas Southwestern Dallas Texas USA; ^19^ Department of Genitourinary Oncology H. Lee Moffitt Cancer Center and Research Institute Tampa Florida USA; ^20^ The James Buchanan Brady Urological Institute and Department of Urology Johns Hopkins University School of Medicine Baltimore Maryland USA; ^21^ Department of Urology Weill Cornell Medical College New York New York USA; ^22^ Department of Urology, Fundació Puigvert Autonomous University of Barcelona Barcelona Spain; ^23^ Department of Cancer Medicine, Gustave Roussy Université Paris‐Saclay Villejuif France; ^24^ Department of Urology Urosud, La Croix Du Sud Hospital Quint‐Fonsegrives France; ^25^ Division of Urology, Department of Special Surgery The University of Jordan Amman Jordan; ^26^ Department of Urology, Second Faculty of Medicine Charles University Prague Czech Republic; ^27^ Department of Urology Semmelweis University Budapest Hungary; ^28^ Research Center for Evidence Medicine, Urology Department Tabriz University of Medical Sciences Tabriz Iran

**Keywords:** cN+, lymph node staging, computed tomography, radical cystectomy, template, urinary bladder neoplasms, urothelial cancer

## Abstract

**Objectives:**

To evaluate whether computed tomography (CT) scans should be performed before or after transurethral resection of bladder tumour (TURBT) for accurate lymph node staging in clinically lymph node‐positive bladder cancer (BCa). Additionally, to identify patient factors that can aid in predicting lymph node metastasis.

**Patients and Methods:**

In this retrospective, multicentre study, we analysed patients with cN+ M0 BCa staged by CT and treated with upfront radical cystectomy (RC) and pelvic lymph node dissection. We stratified patients by the interval between TURBT and CT into three groups: (1) before TURBT; (2) within 30 days after TURBT; and (3) more than 30 days post‐TURBT. Staging accuracy, defined as concordance between clinical and pathological lymph node status, was evaluated. We utilised logistic regression analyses to identify patient factors, including the optimal timing of staging, in predicting pathological lymph node status at RC.

**Results:**

Among 183 patients with cN+ disease, 90 (49%) had pN0 disease at RC. Of these, 40, 36 and 14 were staged before TURBT, within 30 days after TURBT, and more than 30 days post‐TURBT, respectively (*P* = 0.2). Pathological downstaging was most common in cN1 (22%) and cN2 (20%) disease. The overall concordance rate was 23%. The timing of staging did not correlate with pathological lymph node status on logistic regression (all *P* > 0.05). Lymphovascular invasion (LVI) at TURBT was associated with pN status (odds ratio 4.25, confidence interval 2.02–9.34; *P* < 0.001) at RC.

**Conclusion:**

Overall, we found no association between the timing of CT‐based staging and pathological lymph node metastases in cN+ BCa. The data suggest that performing a TURBT prior to staging does not increase the finding of false‐positive nodes on imaging. LVI was the only factor at the time of TURBT associated with pathological lymph node metastasis at RC. Limitations include the multicentre retrospective design and the inclusion of only patients with clinically node‐positive disease.

**Abbreviations**
AUC: area under the receiver‐operating characteristic curve
BCa: bladder cancer
CIS: carcinoma *in situ*
FDG: 18‐fluorodeoxyglucose
IQR: interquartile range
LVI: lymphovascular invasion
MIBC: muscle‐invasive bladder cancer
OR: odds ratio
PLND: pelvic lymph node dissection
RC: radical cystectomy
TURBT: transurethral resection of bladder tumour

## Introduction

Accurate staging of patients with muscle‐invasive bladder cancer (MIBC) is paramount to diagnosis, risk stratification, and subsequent treatment of the underlying disease. For MIBC, major guidelines recommend clinical staging before treatment initiation, with contrast‐enhanced CT as the preferred cross‐sectional imaging modality, given adequate renal function. MRI is an alternative imaging method to CT [1, 2, 3].

The lymphatic spread of clinically lymph node‐positive (cN+) bladder cancer (BCa) is typically identified by enlarged lymph nodes on imaging. Patients identified as node‐positive on imaging often undergo an aggressive multimodal treatment approach to eradicate both visible and non‐visible micro‐metastatic disease. However, up to 50% of cN+ patients are falsely staged as node‐positive when compared with their pathological lymph node status at radical cystectomy (RC) [4, 5, 6]. In clinical practice, many patients undergo imaging after MIBC is detected on transurethral resection of bladder tumour (TURBT). It has been suggested that a short time interval between TURBT and imaging may increase the false‐positive cN+ rate due to a reactive inflammatory, benign pelvic lymph node enlargement caused by TURBT. However, no study has evaluated the impact of the timing of staging relative to TURBT on the concordance between clinical and pathological node stages. Patient factors available at TURBT, such as hydronephrosis, tumour multifocality, and the presence of concomitant carcinoma *in situ* (CIS) or lymphovascular invasion (LVI), might improve the likelihood of identifying true cN+ disease [7, 8, 9].

In the current study, we investigated whether the timing of CT scans relative to TURBT impacts the accuracy of lymph node staging in patients with BCa. We hypothesised that, in patients with cN+ disease, performing CT after TURBT does not increase the false‐positive rate at RC. Additionally, we assessed whether patient factors available at the time of TURBT can aid in predicting true cN+ disease.

## Patients and Methods

### Study Population

This was a retrospective, multicentre study. After institutional review board approval (reference number 1480/2022, Medical University Vienna), we identified patients with cN+ disease treated with upfront RC and pelvic lymph node dissection (PLND) between 1999 and 2021 for cTany N1‐3M0 BCa within the Clinically Positive Lymph Nodes (CLIPOLY) Study Group. We excluded patients with primary metastatic disease (cM1) and patients who received preoperative chemotherapy that could have caused lymph node downstaging. In addition, we excluded patients with fewer than 10 lymph nodes removed at RC and those with missing data regarding staging modality and/or date.

We used the time interval between TURBT and clinical staging for stratification, and patients were categorised as follows: (i) patients who underwent clinical staging before; (ii) within 30 days after; or (iii) more than 30 days after TURBT. If patients had progressive disease, the date of the last TURBT prior to RC was considered. We assumed that the time interval between diagnosis of MIBC at TURBT and RC would not impact the upstaging rate at RC [10]. All patients were staged using abdominopelvic CT, a bone scan (when indicated), and chest CT or radiography within 100 days prior to RC. Locoregional lymph node disease was defined as short‐axis diameter of pelvic lymph nodes >8 mm.

At RC, the extent of PLND (standard or extended) remained at the surgeon's discretion. The PLND templates used followed the recommendations of the European Association of Urology and AUA guidelines at the time of RC [2, 3]. Standard PLND included internal iliac, external iliac as well as obturator lymph nodes. Extended PLND additionally included common iliac and presacral lymph nodes. In each centre, a uropathologist performed a lymph node assessment. Classification of histopathological stages followed the most recent American Joint Committee on Cancer TNM staging system at the time of RC. There was no central imaging or pathology review.

### Patient Characteristics

Clinical characteristics comprised: the time interval (days) between clinical staging and TURBT and the time interval between clinical staging and RC; age at surgery; sex; American Association of Anesthesiologists' physical status score; presence of hydronephrosis at staging; sequence of disease, defined as primary vs progressive BCa; tumour multifocality at TURBT; variant histology at TURBT; concomitant CIS at TURBT; LVI at TURBT; and clinical T and N stage. Surgical and pathological characteristics included: surgical approach (open vs robot‐assisted vs laparoscopic); PLND template; pathological T and N stage; staging accuracy; total lymph node count; number of positive lymph nodes; and lymph node density (positive lymph nodes / lymph nodes removed at RC).

### Study Endpoints and Outcomes

The primary endpoint was assessment of the false‐positive rate of clinical lymph node staging among cN+ patients, comparing the clinical and pathological node stages. False positives were defined as cN+ and pN0. In addition, we assessed the concordance rate (cN+ = pN+), upstaging rate (cN+ < pN+), and downstaging rate (cN+ > pN+). The secondary objective was to assess the association between the time interval from CT to TURBT, as well as patient factors available at TURBT, and histopathological concordance at RC.

### Statistical Analyses

Our statistical analyses were performed in several steps. First, patients were stratified according to the timing of staging (before TURBT, within 30 days after TURBT, more than 30 days after TURBT), and clinical, surgical and pathological characteristics were compared. We report categorical variables as frequencies and proportions, and continuously coded variables with median and interquartile range (IQR). All group comparisons were performed using Fisher's exact test or the chi‐squared test for categorical variables, and the Kruskal–Wallis test for non‐normally distributed continuous variables, as appropriate.

Second, we employed univariable and multivariable logistic regression modelling to test the association between the timing of clinical staging, with regard to TURBT, hydronephrosis, sequence of disease, tumour multifocality at TURBT, variant histology at TURBT, concomitant CIS at TURBT, and LVI at TURBT, and the presence of pathological lymph node metastases at RC.

For the multivariable analyses, we created a reference model adjusted for the effects of common confounders, including age at surgery, sex, clinical T and N stages, and the number of lymph nodes removed at RC. Then, each patient factor as well as the time interval between clinical staging and TURBT, were separately added to the reference model. Ultimately, we evaluated each model's discriminative power in predicting pathological lymph node metastases using the area under the receiver‐operating characteristic curve (AUC) for each model. We compared AUCs using DeLong's test.

Statistical analysis was performed using R version 4.2.1 (R Foundation for Statistical Computing, Vienna, Austria). All tests were two‐sided, and *P* values <0.05 were taken to indicate statistical significance.

## Results

### Patient Characteristics

Overall, we identified 183 patients who underwent preoperative CT staging followed by RC with PLND without preoperative chemotherapy. In total, 77 (42%), 68 (37%) and 38 patients (21%) were staged prior to TURBT, within 30 days after TURBT, and more than 30 days after TURBT, respectively (Table 1). In patients who were staged before TURBT, the median (IQR) time from staging to TURBT was 7 (3–15) days. In patients who underwent clinical staging within 30 days after TURBT, the median time to staging was 13.5 (3–25) days after TURBT. In patients who underwent clinical staging more than 30 days after TURBT, the median time to staging was 45.5 (38–62) days after TURBT.

**Table 1:** Clinical, surgical, and pathological characteristics of 183 patients with clinically lymph node‐positive bladder cancer who underwent staging with CT prior to upfront radical cystectomy.

Characteristic	Overall	Timing of staging			
	*N* = 183	Staging before TURBT, *N* = 77	Staging within 30 days of TURBT, *N* = 68	Staging more than 30 days after TURBT, *N* = 38	*P* value
Age at surgery (continuous), years	67.0 (13.0)	67.0 (16.0)	68.5 (11.0)	66.5 (10.5)	0.7
Sex, *n*/*N* (%)					
Female	46/183 (25)	18/77 (23)	22/68 (32)	6/38 (16)	0.2
Male	137/183 (75)	59/77 (77)	46/68 (68)	32/38 (84)	
ASA physical status, *n*/*N* (%)					
≤2	119/182 (65)	52/77 (68)	44/67 (66)	23/38 (61)	0.8
≥3	63/182 (35)	25/77 (32)	23/67 (34)	15/38 (39)	
Hydronephrosis, *n*/*N* (%)	56/183 (31)	28/77 (36)	16/68 (24)	12/38 (32)	0.2
Sequence of disease, *n*/*N* (%)					
Primary BCa	129/180 (72)	58/77 (75)	49/68 (72)	22/35 (63)	0.4
Progressive BCa	51/180 (28)	19/77 (25)	19/68 (28)	13/35 (37)	
Tumour multifocality at TURBT, *n*/*N* (%)	65/180 (36)	33/77 (43)	22/66 (33)	10/37 (27)	0.2
Variant histology at TURBT, *n*/*N* (%)	34/183 (19)	18/77 (23)	10/68 (15)	6/38 (16)	0.4
Concomitant CIS at TURBT, *n*/*N* (%)	48/182 (26)	22/76 (29)	18/68 (26)	8/38 (21)	0.7
LVI at TURBT, *n*/*N* (%)	53/175 (30)	22/74 (30)	21/67 (31)	10/34 (29)	>0.9
Time interval between TURBT and staging, days	15.0 (26.0)	−7.0 (12.0)	+13.5 (22.0)	+45.5 (23.8)	**<0.001**
Time interval between staging and RC, days	34.0 (41.5)	45.0 (41.0)	27.5 (36.8)	20.0 (24.0)	**<0.001**
Clinical T stage *n*/*N* (%)					
<cT2	19/183 (10)	5/77 (6.5)	5/68 (7.4)	9/38 (24)	**0.023**
cT2	93/183 (51)	44/77 (57)	30/68 (44)	19/38 (50)	
≥cT3	71/183 (39)	28/77 (36)	33/68 (49)	10/38 (26)	
Clinical N stage *n*/*N* (%)					
cN1	89/183 (49)	36/77 (47)	38/68 (56)	15/38 (39)	0.3
cN2	64/183 (35)	28/77 (36)	18/68 (26)	18/38 (47)	
cN3	30/183 (16)	13/77 (17)	12/68 (18)	5/38 (13)	
Surgical approach at RC *n*/*N* (%)					
LRC	1/182 (0.5)	1/77 (1.3)	0/68 (0)	0/37 (0)	NA
Open	180/182 (99)	76/77 (99)	67/68 (99)	37/37 (100)	
RARC	1/182 (0.5)	0/77 (0)	1/68 (1.5)	0/37 (0)	
PLND template *n*/*N* (%)					
Standard	47/183 (26)	17/77 (22)	18/68 (26)	12/38 (32)	0.5
Extended	136/183 (74)	60/77 (78)	50/68 (74)	26/38 (68)	
Pathological T stage *n*/*N* (%)					
<pT2	39/183 (21)	11/77 (14)	22/68 (32)	6/38 (16)	**0.033**
pT2	41/183 (22)	15/77 (19)	17/68 (25)	9/38 (24)	
≥pT3	103/183 (56)	51/77 (66)	29/68 (43)	23/38 (61)	
Pathological N stage *n*/*N* (%)					
pN0	90/183 (49)	40/77 (52)	36/68 (53)	14/38 (37)	0.2
pN+	93/183 (51)	37/77 (48)	32/68 (47)	24/38 (63)	
Staging accuracy *n*/*N* (%)					
Concordance	42/183 (23)	17/77 (22)	12/68 (18)	13/38 (34)	0.3
Downstaging	103/183 (56)	44/77 (57)	43/68 (63)	16/38 (42)	
Upstaging	38/183 (21)	16/77 (21)	13/68 (19)	9/38 (24)	
Number of lymph nodes removed at RC	27.0 (16.5)	27.0 (15.0)	25.5 (20.0)	26.5 (18.5)	0.7
Lymph node density	2.3 (11.1)	0.0 (7.1)	0.0 (9.8)	8.8 (21.9)	**0.034**

Values are median (interquartile range), unless otherwise stated. Kruskal–Wallis rank sum test; Pearson's chi‐squared test; Fisher's exact test. Bold formatting indicates *P* values < 0.05. Lymph node density is defined as positive lymph nodes / lymph nodes removed at RC. ASA, American Society of Anesthesiologists; CIS, carcinoma *in situ*; LN, lymph nodes; LRC, laparoscopic radical cystectomy; LVI, lymphovascular invasion; NA, not applicable; PLND, pelvic lymph node dissection; RARC, robot‐assisted radical cystectomy; RC, radical cystectomy; TURBT, transurethral resection of bladder tumour.

In patients who were staged before TURBT, the median (IQR) time between clinical staging and RC was 45 (27–68) days. In patients who underwent clinical staging within 30 days after TURBT, the median time between clinical staging and RC was 27.5 (16–52) days. In patients who underwent clinical staging more than 30 days after TURBT, the median time between clinical staging and RC was 20 (8–32) days (Table 1).

### Staging and Pathological Outcomes

In the overall cohort, 89 (49%), 64 (35%) and 30 patients (16%) had cN1, cN2 and cN3 disease, respectively, and 164 (90%) had muscle‐invasive disease according to clinical staging (Table 1). Overall, 47 (26%) and 136 patients (74%) underwent standard and extended PLND, respectively. The median (IQR) number of lymph nodes removed was 27 (18.5–35) across all patients. At RC, the false‐positive rate was 49% (90/183 patients had pN0 disease). Of these patients, 40, 36 and 14 were staged prior to TURBT, within 30 days after TURBT, and more than 30 days after TURBT, respectively (*P* = 0.2; Table 1). In pN0 patients, the median (IQR) number of lymph nodes removed was 27 (19–37).

**Fig. 1:**
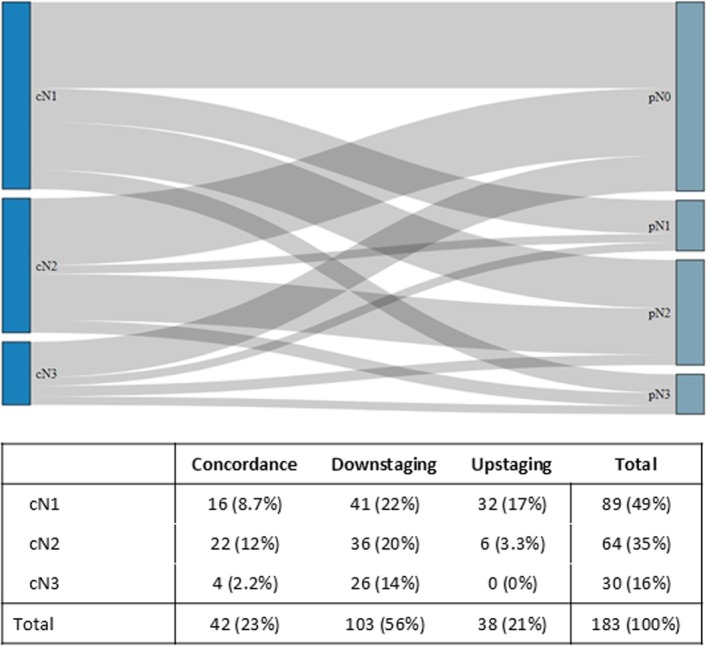
Sankey diagram as well as concordance, downstaging, and upstaging in 183 patients with clinically lymph node‐positive bladder cancer who underwent staging with CT prior to radical cystectomy.

Across all clinical and pathological N stages, the concordance rate was 23% (Table 1). In patients staged before TURBT and within 30 days after TURBT, the concordance rate was 22% and 18%, respectively (*P* = 0.3). Comparing the clinical and pathological node stages, the concordance rate was highest in patients with cN2 disease (34% [22/64]; Fig. 1). Downstaging was most common in cN1 (41/183 [22%]) and cN2 disease (36/183 [20%]). Upstaging from cN1 occurred in 32/183 patients (17%; Fig. 1).

### Univariable and Multivariable Logistic Regression Analyses

The results of the logistic regression analyses are displayed in Tables 2 and S1. On univariable analysis, the different intervals between staging and TURBT were not associated with pathologically confirmed lymph node metastases at RC (staging within 30 days after TURBT: odds ratio [OR] 0.96, CI 0.5–1.85, *P* = 0.9; staging more than 30 days after TURBT: OR 1.85, CI 0.84–4.18, *P* = 0.1). We found similar results when accounting for the effects of various patient factors available at TURBT (staging within 30 days after TURBT: OR 0.91, CI 0.42–1.96, *P* = 0.8; staging more than 30 days after TURBT: OR 1.93, CI 0.75–5.17, *P* = 0.2 [Table 2]). On multivariable analysis, only LVI at TURBT was associated with pN stage (OR 3.84, CI 1.75–8.81, *P* = 0.001 [Table 2]).

**Table 2:** Univariable and multivariable logistic regression analyses predicting the pathological lymph node status in 183 patients with clinically lymph node‐positive bladder cancer who underwent staging with CT prior to radical cystectomy.

	Univariable model			Multivariable all factors			Multivariable timing only		
	OR	95% CI	*P* value	OR	95% CI	*P* value	OR	95% CI	*P* value
Age at surgery (continuous)	0.98	0.95, 1.01	0.14	0.97	0.94, 1.00	**0.032**	0.98	0.95, 1.01	0.15
Sex (reference: female)	0.58	0.29, 1.14	0.12	0.83	0.34, 2.04	0.7	0.57	0.27, 1.20	0.14
Clinical T stage (reference: <cT2)									
cT2	0.84	0.31, 2.28	0.7	1.11	0.31, 4.08	0.9	1.09	0.34, 3.52	0.9
≥cT3	1.04	0.37, 2.87	0.9	1.54	0.41, 5.81	0.5	1.52	0.45, 5.15	0.5
Clinical N stage (reference: cN1)									
cN2	0.85	0.45, 1.63	0.6	0.57	0.25, 1.24	0.2	0.74	0.36, 1.50	0.4
cN3	0.65	0.28, 1.50	0.3	0.66	0.25, 1.69	0.4	0.64	0.26, 1.59	0.3
Number of LNs removed at RC (continuous)	0.99	0.97, 1.01	0.2	0.99	0.97, 1.01	0.5	0.99	0.97, 1.01	0.3
Time interval between staging and TURBT (reference: staging before TURBT)									
Within 30 days after TURBT	0.96	0.50, 1.85	0.9	0.91	0.42, 1.96	0.8	0.79	0.39, 1.58	0.5
After >30 days after TURBT	1.85	0.84, 4.18	0.13	1.93	0.75, 5.17	0.2	1.79	0.74, 4.47	0.2
Hydronephrosis (reference: no)	1.17	0.62, 2.21	0.6	1.83	0.85, 4.06	0.13			
Sequence of disease (reference: primary BCa)	1.28	0.67, 2.46	0.5	1.56	0.69, 3.55	0.3			
Tumour multifocality (reference: no)	1.71	0.93, 3.18	0.09	1.44	0.69, 2.99	0.3			
Variant histology (reference: no)	1.73	0.81, 3.78	0.16	1.54	0.61, 3.95	0.4			
Concomitant CIS (reference: no)	0.67	0.34, 1.30	0.2	0.98	0.45, 2.15	>0.9			
LVI (reference: no)	3.77	1.91, 7.77	**<0.001**	3.84	1.75, 8.81	**0.001**			

Bold formatting indicates *P* values <0.05. CIS, carcinoma *in situ*; LN, lymph node; LVI, lymphovascular invasion; OR, odds ratio; RC, radical cystectomy; TURBT, transurethral resection of bladder tumour.

When added to a reference model, we found no association between the timing of staging and pathological node stage at RC (staging within 30 days after TURBT: OR 0.79, CI 0.39–1.58, *P* = 0.5; staging more than 30 days after TURBT: OR 1.79, CI 0.74–4.47, *P* = 0.2 [Table 2]). The AUC for diagnostic testing was 0.64 (95% CI 0.56–0.73, DeLong's test *P* = 0.3). Similarly, the presence of hydronephrosis at staging, sequence of disease (primary vs progressive BCa), tumour multifocality at TURBT, variant histology at TURBT, and CIS at TURBT were not associated with pathological lymph node status when added to a reference model (all *P* > 0.05; Table S1) and did not improve the predictive ability of each model (DeLong's test *P* > 0.05; Fig. S1). Adding LVI to the reference model, LVI was associated with pathological lymph node status (OR 4.25, CI 2.02–9.34, *P* < 0.001) and improved the reference model's discriminative ability (AUC 0.74, 95% CI 0.66–0.81, DeLong's test *P* = 0.01 [Table S1 and Fig. S1]).

## Discussion

Optimising treatment strategies for patients with MIBC strongly relies on accurate staging, particularly lymph node staging. In this study, we assessed whether identifying true‐positive lymph node metastases at RC depends on whether CT‐based clinical staging is performed before or after TURBT. The underlying rationale was that a short time interval between TURBT and clinical staging might cause a reactive, benign inflammatory enlargement of pelvic lymph nodes, consequently detected as false‐positive cN+ disease. However, we found no association between the interval from CT‐based lymph node staging and TURBT and the identification of pathological lymph node metastases at RC in cN+ BCa. This suggests that CT staging performed within the first 30 days after TURBT does not increase the false‐positive rate for lymph node metastasis. Indeed, almost 50% of our cN+ patients had pN0 disease at RC, regardless of whether clinical staging was performed before TURBT or within 30 days after TURBT. We found no previous study that has addressed this clinically relevant question.

Next, we asked whether patient factors available at TURBT indicating biologically aggressive disease may aid in improving the identification of true cN+ disease. LVI was the only factor at the time of TURBT that improved the prediction of pathological lymph node metastasis at RC on logistic regression analyses, despite adjusting for the effects of confounding factors. However, we did not incorporate radiological lymph node features, which could potentially improve the identification of true cN+ disease [11, 12].

Our results confirm a significant staging bias in identifying patients with cN+ disease [4, 5]. However, in our study, we selected patients carefully to avoid false‐positive staging results. Indeed, none of the patients included in this study received preoperative systemic therapy. Moreover, almost three quarters of the patients underwent extended PLND, and the median number of lymph nodes removed in the full cohort and in the pN0 population was 27, suggesting that a sufficient number of lymph nodes were removed to detect pathological lymph node metastases. In addition, we excluded patients with fewer than 10 lymph nodes removed at RC to ensure adequate pathological lymph node staging.

While contrast‐enhanced CT is still the guideline‐endorsed staging modality, one may argue that our study was limited by using a single modality (CT) during staging. With the advent of more modern staging technologies, MRI, with its superior soft‐tissue contrast resolution, has been increasingly investigated. However, MRI failed to show a meaningful benefit in clinical lymph node staging compared to CT alone [2, 13, 14, 15]. In summary, both modalities (CT and MRI) are limited by their low sensitivity and specificity, in particular, in detecting lymph node metastases, despite the advantages of MRI regarding locoregional staging of the bladder [1, 15, 16]. Moreover, both imaging modalities primarily rely on structural changes, with lymph node size being the key factor, using an arbitrary cut‐off.

In contrast, the advantage of PET‐CT/MRI is that it captures metabolic activity and morphological changes, providing structural and functional information of the underlying disease. While 18‐fluorodeoxyglucose (FDG) is the most common tracer, to date, FDG‐PET failed to show significant improvement in the staging accuracy during primary lymph node staging and the assessment of treatment response after systemic therapy, in particular, due to its low specificity [15, 17]. However, it can lead to upstaging from cN0 to cN+ disease in approximately one fifth of the patients compared to CT alone, and it may provide additional information regarding distant metastases, thereby influencing the clinical management of MIBC patients [18, 19, 20, 21]. Nevertheless, its impact on the survival of patients with cN+ BCa remains elusive [20]. While novel tracers are being tested and further studies are ongoing, due to the current lack of high‐quality data, there is no recommendation for the routine use of FDG‐PET/CT for lymph node staging in cN+ MIBC [2, 22, 23].

Patients’ lymph node status at RC is an important prognostic indicator, influencing the treatment approach, survival rates, and follow‐up and surveillance strategies post‐treatment. In contrast, cN status is inherently inadequate, leading to a high false‐positive rate, potentially resulting in unnecessary treatment intensification. Accurate staging is particularly critical for patients with cN1 BCa, who are more likely to achieve curative outcomes using the combination of systemic and local therapy, compared to those with cN2–3 disease who have a significantly poorer prognosis despite aggressive treatment [24, 25].

Our study's results must be interpreted with consideration of the multicentric, retrospective study design, its inherent limitations, and the sample size, which leaves a residual risk that the study was underpowered to detect statistically significant differences. First, the stratification factor used for the time interval between TURBT and clinical staging was arbitrary, as there are limited data on the duration and extent to which TURBT may cause reactive pelvic lymph node enlargement detected as false‐positive cN+ disease. Second, there was no central radiological and pathological review. Third, the quality and readability of CT imaging improved over the study period, a factor we could not control for. Fourth, by focusing solely on patients with cN+ disease, we did not assess sensitivity and specificity. However, we purposely excluded patients with cN0 disease as numerous studies have previously addressed this question [15]. Fifth, the absence of follow‐up imaging limits our ability to fully exclude residual nodal disease after PLND.

Despite these limitations, our study demonstrates that the timing of staging relative to TURBT did not affect the concordance between clinical and pathological lymph node metastases in cN+ BCa, supporting the immediate use of CT‐based staging post‐TURBT. Moreover, patient factors available at TURBT linked to biologically aggressive disease did not improve the prediction of pathological lymph node metastases at RC. While the sensitivity and specificity of MRI in detecting lymph node metastases vary significantly, and newer methods are not yet widely available, contrast‐enhanced CT remains the standard staging tool for MIBC, despite the inherent false‐positive rate.

## Disclosure of Interests

None.

## Supporting information


**Fig. S1.** Area under the receiver operating curve (AUC) for separate multivariable logistic regression models predicting pathological lymph node metastases in 183 patients with clinically lymph node‐positive bladder cancer who underwent staging with computed tomography prior to radical cystectomy. CIS, carcinoma *in situ*; LVI, lymphovascular invasion; VH, variant histology.

**Table S1.** Multivariable logistic regression models assessing the association between clinical factors available at TURBT and the pathological lymph node status in 183 patients with clinically lymph node‐positive bladder cancer who underwent staging with computed tomography prior to radical cystectomy.

## Appendix A CLIPOLY study group collaborators

Luca Antonelli (Department of Urology, Luzerner Kantonsspital, Luzern, Switzerland), Mara Bacchiani (Unit of Oncologic Minimally‐Invasive Urology and Andrology, Department of Experimental and Clinical Medicine, Careggi Hospital, University of Florence, Florence, Italy), Ronan Flippot (Department of Cancer Medicine, Gustave Roussy, Université Paris‐Saclay, Villejuif, France), Abhinav Khanna (Department of Urology, Mayo Clinic, Rochester, Minnesota, USA), Elisabeth Maier (Department of Urology, München Klinik Bogenhausen, Munich, Germany), Gautier Marcq (Department of Urology, Claude Huriez Hospital, CHU Lille, Lille, France, University of Lille, CNRS, Inserm, CHU Lille, Institut Pasteur de Lille, UMR9020‐U1277 ‐ CANTHER ‐ Cancer Heterogeneity Plasticity and Resistance to Therapies, Lille, France), Andrea Minvervini (Unit of Oncologic Minimally‐Invasive Urology and Andrology, Department of Experimental and Clinical Medicine, Careggi Hospital, University of Florence, Florence, Italy), Keiichiro Mori (Department of Urology, The Jikei University School of Medicine, Tokyo, Japan), Maximilian Pallauf (The James Buchanan Brady Urological Institute and Department of Urology, Johns Hopkins University School of Medicine, Baltimore, MD, USA; Department of Urology, University Hospital Salzburg, Paracelsus Medical University, Salzburg, Austria), Nicolas Penel (Lille University and Department of Medical Oncology, Centre Oscar‐Lambret, Lille, France), Julien Sarkis (Service d'Urologie, Hôpital Erasme, Université Libre de Bruxelles, Bruxelles, Belgium), Francesco Soria (Division of Urology, Department of Surgical Sciences, San Giovanni Battista Hospital, University of Studies of Torino, Turin, Italy), Guus A.H. Tendijck (Department of Urology, The Netherlands Cancer Institute, Amsterdam, The Netherlands), Noor van Ginkel (Department of Urology, The Netherlands Cancer Institute, Amsterdam, The Netherlands), Solomon Woldu (Department of Urology, University of Texas Southwestern, Dallas, Texas, USA).
