# Supplementary figures and images for: Impact of timing of computed tomography staging and patient factors on the detection of ‘true’ cN+ bladder cancer
Source: BJU Int. 2025 Jul 9;136(5):911–9. doi: 10.1111/bju.16851 (PMC12522521; doi:10.1111/bju.16851)

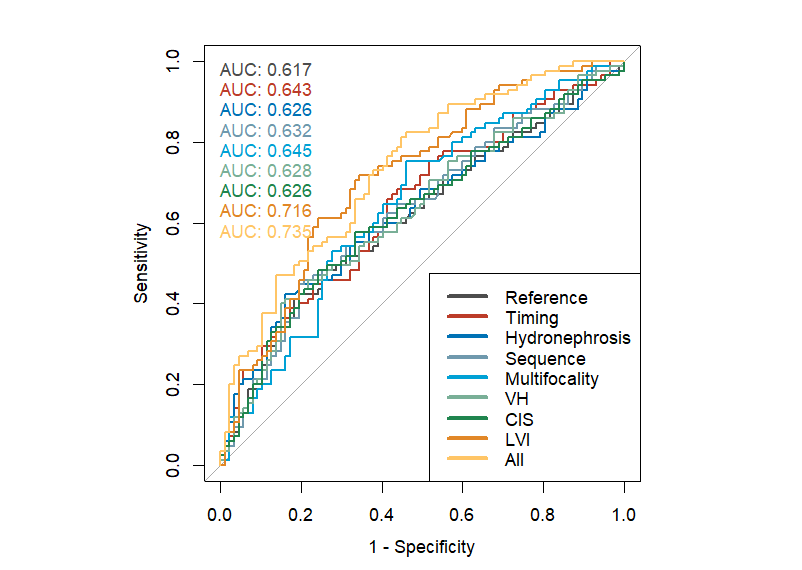

Supplement: Supplementary file 1 — Fig. S1. Area under the receiver operating curve (AUC) for separate multivariable logistic regression models predicting pathological lymph node metastases in 183 patients with clinically lymph node‐positive bladder cancer who underwent staging with computed tomography prior to radical cystectomy. CIS, carcinoma in situ; LVI, lymphovascular invasion; VH, variant histology. [file BJU-136-911-s001.tiff]
