# Supplementary material for: Impact of timing of computed tomography staging and patient factors on the detection of ‘true’ cN+ bladder cancer
Source: BJU Int. 2025 Jul 9;136(5):911–9. doi: 10.1111/bju.16851 (PMC12522521; doi:10.1111/bju.16851)
Supplement: Supplementary file 2 — Table S1. Multivariable logistic regression models assessing the association between clinical factors available at TURBT and the pathological lymph node status in 183 patients with clinically lymph node‐positive bladder cancer who underwent staging with computed tomography prior to radical cystectomy. [file BJU-136-911-s002.docx]

| **Supplementary Table 1:** Multivariable logistic regression models assessing the association between clinical factors available at TURBT and the pathological lymph node status in 183 patients with clinically lymph node-positive bladder cancer who underwent staging with computed tomography prior to radical cystectomy. | | | | | | | | | | | | |
| --- | --- | --- | --- | --- | --- | --- | --- | --- | --- | --- | --- | --- |
|  | **Reference model** | | | **Hydronephrosis** | | | **Sequence of disease** | | | **Tumor multifocality** | | |
|  | **OR** | **95% CI** | **p-value** | **OR** | **95% CI** | **p-value** | **OR** | **95% CI** | **p-value** | **OR** | **95% CI** | **p-value** |
| Age at surgery (continuous) | 0.98 | 0.95, 1.01 | 0.13 | 0.98 | 0.95, 1.00 | 0.1 | 0.98 | 0.95, 1.00 | 0.11 | 0.98 | 0.95, 1.00 | 0.11 |
| Sex (ref: female) | 0.63 | 0.30, 1.31 | 0.2 | 0.60 | 0.29, 1.25 | 0.2 | 0.58 | 0.27, 1.21 | 0.2 | 0.68 | 0.32, 1.43 | 0.3 |
| Clinical T-stage (ref: <cT2) |  |  |  |  |  |  |  |  |  |  |  |  |
| cT2 | 0.96 | 0.30, 3.03 | >0.9 | 1.06 | 0.33, 3.35 | >0.9 | 1.09 | 0.34, 3.55 | 0.9 | 0.99 | 0.31, 3.16 | >0.9 |
| ≥cT3 | 1.22 | 0.37, 3.97 | 0.7 | 1.34 | 0.41, 4.39 | 0.6 | 1.31 | 0.40, 4.32 | 0.7 | 1.22 | 0.37, 4.04 | 0.7 |
| Clinical N-stage (ref: cN1) |  |  |  |  |  |  |  |  |  |  |  |  |
| cN2 | 0.85 | 0.43, 1.69 | 0.6 | 0.78 | 0.38, 1.57 | 0.5 | 0.83 | 0.42, 1.65 | 0.6 | 0.86 | 0.43, 1.70 | 0.7 |
| cN3 | 0.66 | 0.26, 1.62 | 0.4 | 0.65 | 0.26, 1.61 | 0.4 | 0.65 | 0.26, 1.59 | 0.3 | 0.65 | 0.26, 1.62 | 0.4 |
| Number of LN removed at RC (continuous) | 0.99 | 0.97, 1.01 | 0.3 | 0.99 | 0.97, 1.01 | 0.3 | 0.99 | 0.97, 1.01 | 0.4 | 0.99 | 0.97, 1.01 | 0.4 |
| Time interval between staging and TURBT (ref: staging before TURBT) |  |  |  |  |  |  |  |  |  |  |  |  |
| Hydronephrosis (ref: no) |  |  |  | 1.57 | 0.79, 3.17 | 0.2 |  |  |  |  |  |  |
| Sequence of disease (ref: primary BCa) |  |  |  |  |  |  | 1.50 | 0.73, 3.15 | 0.3 |  |  |  |
| Tumor multifocality (ref: no) |  |  |  |  |  |  |  |  |  | 1.68 | 0.88, 3.24 | 0.12 |
| Variant histology (ref: no) |  |  |  |  |  |  |  |  |  |  |  |  |
| Concomitant CIS (ref: no) |  |  |  |  |  |  |  |  |  |  |  |  |
| LVI (ref: no) |  |  |  |  |  |  |  |  |  |  |  |  |
| CI = confidence interval; CIS = carcinoma in situ; LN = lymph nodes; LVI = lymphovascular invasion; OR = odds ratio; RC = radical cystectomy; TURBT = transurethral resection of bladder tumor | | | | | | | | | | | | |
| Bold letters indicate p-value <0.05 | | | | | | | | | | | | |

| **Supplementary Table 1 continued.** | | | | | | | | | |
| --- | --- | --- | --- | --- | --- | --- | --- | --- | --- |
|  | **Variant histology** | | | **CIS** | | | **LVI** | | |
|  | **OR** | **95% CI** | **p-value** | **OR** | **95% CI** | **p-value** | **OR** | **95% CI** | **p-value** |
| Age at surgery (continuous) | 0.98 | 0.95, 1.01 | 0.2 | 0.98 | 0.95, 1.01 | 0.13 | 0.97 | 0.94, 1.00 | **0.046** |
| Sex (ref: female) | 0.73 | 0.34, 1.55 | 0.4 | 0.66 | 0.31, 1.37 | 0.3 | 0.93 | 0.42, 2.07 | 0.9 |
| Clinical T-stage (ref: <cT2) |  |  |  |  |  |  |  |  |  |
| cT2 | 0.90 | 0.28, 2.83 | 0.9 | 0.93 | 0.29, 2.94 | 0.9 | 0.74 | 0.22, 2.44 | 0.6 |
| ≥cT3 | 1.12 | 0.34, 3.64 | 0.9 | 1.16 | 0.35, 3.81 | 0.8 | 1.06 | 0.31, 3.62 | >0.9 |
| Clinical N-stage (ref: cN1) |  |  |  |  |  |  |  |  |  |
| cN2 | 0.80 | 0.40, 1.60 | 0.5 | 0.84 | 0.42, 1.67 | 0.6 | 0.80 | 0.38, 1.63 | 0.5 |
| cN3 | 0.65 | 0.25, 1.59 | 0.3 | 0.66 | 0.26, 1.62 | 0.4 | 0.71 | 0.28, 1.79 | 0.5 |
| Number of LN removed at RC (continuous) | 0.99 | 0.97, 1.01 | 0.3 | 0.99 | 0.97, 1.01 | 0.3 | 0.99 | 0.97, 1.02 | 0.5 |
| Time interval between staging and TURBT (ref: staging before TURBT) |  |  |  |  |  |  |  |  |  |
| Hydronephrosis (ref: no) |  |  |  |  |  |  |  |  |  |
| Sequence of disease (ref: primary) |  |  |  |  |  |  |  |  |  |
| Tumor multifocality (ref: no) |  |  |  |  |  |  |  |  |  |
| Variant histology (ref: no) | 1.69 | 0.72, 4.07 | 0.2 |  |  |  |  |  |  |
| CIS (ref: no) |  |  |  | 0.78 | 0.38, 1.59 | 0.5 |  |  |  |
| LVI (ref: no) |  |  |  |  |  |  | 4.25 | 2.02, 9.34 | **<0.001** |
| CI = confidence interval; CIS = carcinoma in situ; LN = lymph nodes; LVI = lymphovascular invasion; OR = odds ratio; RC = radical cystectomy; TURBT = transurethral resection of bladder tumor | | | | | | | | | |
| Bold letters indicate p-value <0.05 | | | | | | | | | |
